# Do pain management apps use evidence-based psychological components? A systematic review of app content and quality

**DOI:** 10.1080/24740527.2022.2030212

**Published:** 2022-06-03

**Authors:** Megan MacPherson, A. Myfanwy Bakker, Koby Anderson, Susan Holtzman

**Affiliations:** a School of Health and Exercise Sciences, University of British Columbia,Okanagan Campus; 3333 University Way, Kelowna, BC, V1V 1V, Canada; b Department of Psychology, Universiy of British Columbia, Kelowna, British Columbia, Canada

**Keywords:** smartphone, telemedicine, mobile applications, chronic pain, pain management, psychosocial intervention

## Abstract

**Background:**

With hundreds of pain management apps on the Canadian marketplace, it can be challenging for patients and clinicians to select effective and evidence-based mobile health (mHealth) apps that address pain from a biopsychosocial perspective.

**Aims:**

The aim of this study is to identify pain management apps within the Canadian app marketplaces to aid clinicians in recommending apps.

**Methods:**

The iOS and Android marketplaces were systematically searched to identify pain management apps that included at least one core component of cognitive behavioral therapy (CBT) or mindfulness- and acceptance-based therapies. Selected apps were assessed using a researcher developed psychological components checklist, and the Mobile App Rating Scale (MARS). These two measures provided a robust assessment of the apps’ technical abilities and psychological principles being implemented.

**Results:**

Five hundred eight pain management apps were identified, yet only 12 included a psychological component and were available for evaluation. On average, apps contained 8.10 out of 18 psychological components (SD = 2.77) with a MARS quality rating of 4.02 out of 5 (SD = 0.32). The most common psychological components were grounded in CBT, including psychoeducation, sleep hygiene, behavioral activation, coping skills training, and social support. Among the least commonly included components were goal setting, values, and culture/diversity. Two-thirds of the apps involved health care practitioners in their development, but independent scientific review of apps was scarce.

**Conclusion:**

The highest scoring apps (Curable, Pathways, Vivify) are highlighted for health care practitioners who may wish to recommend mHealth technologies to their patients for pain management. Future directions for research and app development are discussed.

## RÉSUMÉ

**Contexte**: Le fait qu’il existe des centaines d'applications de prise en charge de la douleur sur le marché canadien peut faire en sorte qu’il soit difficile pour les patients et les cliniciens de choisir une application de santé mobile (mHealth) efficace et fondée sur des données probantes, qui aborde la douleur d'un point de vue biopsychosocial.

**But**: Le but de cette étude est de répertorier les applications de prise en charge de la douleur disponibles sur les marchés des applications canadiens afin d'aider les cliniciens à en recommander certaines.

**Méthodes**: Les marchés iOS et Android ont été systématiquement consultés pour répertorier les applications de prise en charge de la douleur qui comprennent au moins une composante de base de la thérapie cognitivo-comportementale (TCC) ou des thérapies fondées sur la pleine conscience et l'acceptation. Les applications sélectionnées ont été évaluées à l'aide d'une liste de vérification des composantes psychologiques élaborée par un chercheur et le Mobile App Rating Scale (MARS). Ces deux mesures ont fourni une évaluation robuste des capacités techniques et des principes psychologiques mis en œuvre.

**Résultats**: Cinq cent huit applications de prise en charge de la douleur ont été répertoriées, mais seulement 12 d’entre elles comprenaient une composante psychologique et étaient disponibles pour l'évaluation. En moyenne, les applications contenaient 8,10 des 18 composantes psychologiques (ÉT = 2,77) et obtenaient un score de qualité MARS de 4,02 sur 5 (ÉT = 0,32. Les composantes psychologiques les plus courantes étaient ancrées dans la TCC, y compris la psychoéducation, l'hygiéne du sommeil, l'activation comportementale, la formation aux compétences d'adaptation et le soutien social. Les composantes les moins souvent incluses étaient l'établissement d'objectifs, les valeurs et la culture/diversité. Deux-tiers des applications avaient impliqué des praticiens de la santé dans leur développement, mais peu d’entre elles avaient été soumises à un examen scientifique indépendant.

**Conclusion**: Les applications ayant obtenu les scores les plus élevés (Curable, Pathways, Vivify) sont mises en évidence à l’intention des praticiens des soins de santé qui peuvent souhaiter recommander des technologies de santé mobile à leurs patients pour la prise en charge de la douleur. Les orientations futures pour la recherche et le développement d'applications sont abordées.

## Introduction

More than 7.6 million Canadians over the age of 15 experience chronic pain, with associated costs of $40 billion in 2019.^1^ Chronic pain is one of the most common reasons for seeking medical care in Canada,^2^ and it is a leading cause of disability globally.^3^ The biopsychosocial model of chronic pain is considered to be the most appropriate therapeutic approach for managing this condition.^4^ Futhermore, rates of mental health difficulties are substantially higher among those with chronic pain compared to the general population, with up to 65% and 85% reporting clinically significant levels of anxiety and depression, respectively.^5,6^ As such, addressing psychological factors in the treatment for chronic pain is paramount. Current evidence-based psychological interventions for people with chronic pain, as well as those with comorbid mental health conditions, include cognitive behavioral therapy (CBT) and mindfulness- and acceptance-based therapies, such as mindfulness-based stress reduction and acceptance and commitment therapy (ACT).^7–11^ Despite a strong body of research supporting the effectiveness of these therapeutic approaches,^12–15^ patients may encounter systemic barriers to accessing them, including long waitlists, financial costs, and the physical and logistical challenges of attending weekly therapy appointments.^16,17^ Given the enormous burden of chronic pain on patients, families, health care systems, and society,^18–20^ there is a pressing need for effective, affordable, and easily deployable pain management interventions.

Telehealth, which includes the provision of health care services though mobile phone technologies (mHealth) and video conferencing, increased substantially in Canada in the year 2020, largely in response to the COVID-19 pandemic. For example, in Ontario, the number of physicians who reported providing telehealth appointments increased from 7% in 2019 to 86% in 2020.^21^ Further, a survey of 1800 Canadians highlighted that approximately half of all respondents accessed health advice via their phones, e-mail, text messaging, or videoconferencing during the year 2020 and believe that expanding telehealth access beyond COVID-19 would benefit patients.^22^

Within the context of chronic pain, there is a critical need to incorporate mHealth into the provision of clinical care, which can include virtual medical visits and referrals to online health resources for pain self-management.^23–26^ Given the widespread adoption of smartphone devices,^27^ smartphone applications (apps) have the potential to play a unique and powerful role in improving access to evidence-based psychological interventions for chronic pain.^12–15^ However, despite hundreds of pain apps on the market, there is little guidance as to which ones may be most effective and based on current best practices. Consequently, selecting the right app can prove time-consuming and potentially lead to adverse patient outcomes if not vetted correctly.^28,29^

A number of reviews of chronic pain self-management apps have been completed over the last decade.^13,30–38^ Self-management interventions typically address a wide range of topics, including goal setting, nutrition, relaxation, and pacing, with the goal of helping patients better manage their symptoms in daily life.^39,40^ However, for the large number of patients who struggle with comorbid mental health problems, a more intensive, targeted approach is often required to adequately address psychological symptoms and distress. In traditional in-person settings, CBT and ACT for pain and comorbid mental health conditions typically involves eight to ten sessions of individual or group therapy led by a mental health professional. These approaches have been successfully adapted to online contexts, including guided and automated Internet-based interventions, video conferencing, and telephone-administered interventions.^41–44^ However, the extent to which evidence-based psychotherapies are currently available to patients via pain management apps (which could offer greater useability and portability) remains unclear.

Despite the recent swell of published reviews assessing the quality of pain management apps, few have specifically evaluated the inclusion of core psychological components found in CBT and mindfulness- and acceptance-based interventions. Lalloo and colleagues^13^ evaluated the self-management functions on 279 pain management apps available in Canadian app stores and found that the majority (77.4%) offered training in at least some type of pain self-care (e.g., relaxation and distraction techniques), and almost half (45.9%) offered basic pain education. Portelli and Eldred^38^ conducted a more detailed evaluation of the psychological components included in pain management apps available in the United States and found only six apps that employed specific CBT principles. However, neither of these reviews examined mindfulnesss- or acceptance-based strategies, and given that the searches were conducted in 2014, the findings no longer capture the current state of chronic pain management apps. More recently, Devan and colleagues^30^ published a review of pain self-management apps based on a search of New Zealand app stores conducted in 2018. The review was strengthened by using the Mobile App Rating Scale (MARS), a widely used, standardized tool to assess app quality^45^; yet, again, there was limited attention to core components of acceptance- and mindfulness-based approaches for pain. Further, only 11 of the 19 apps were specifically intended for use in pain populations, and only 5 of those are available to Canadian users.

Given the urgent and pervasive need for greater access to mental health supports for people living with chronic pain, this research offers a systematic review of CBT and mindfulness- and acceptance-based therapies currently available in Canadian app marketplaces targeting pain management. The first aim was to characterize the specific nature of the psychological components featured on these apps. The second aim was to evaluate the technical abilities and overall quality of these apps using the MARS. The overarching goals were to assist pain practictioners in making evidence-based decisions when recommending mobile apps to their patients, including those with comorbid mental health challenges, and to identify targets for future research and app development in this rapidly advancing field.

## Methods

### Search Procedure

Our search for relevant apps was conducted within the Android and Apple iOS app marketplaces given that together they account for 99.2% of the global smartphone market share.^46^ iPhones running the most recent version of iOS (version 13) were used to conduct the search of the Apple App Store. Android Studio^47^ (a computer software) was used to simulate an Android device and provide the researchers full access to the Google Play store. For our search, the Google Pixel 3 smartphone was emulated with the latest available version of Android (version 10).

In October 2020, we searched the Canadian Apple and Google Play stores using the following independent search terms: “pain,” “chronic pain,” and “pain management.” Apps that were released prior to January 2014 were removed to avoid redundancy with Portelli and Eldred’s^38^ previous review. To be included in the current study, the app was first required to (1) include the word “pain” in the app description, (2) be aimed at individuals experiencing pain, (3) be specific to pain management (i.e., content and interventions were framed in the context of pain management), (4) be interactive, and (5) be presented in English. Apps were excluded if they (1) provided information without any interactive components (i.e., only informational text and images, without activities to actively engage the user), (2) were designed for patients being seen by a specific pain clinic, or (3) targeted caregivers or health care practitioners.

Following this initial screening, remaining apps were screened by two independent reviewers (both senior doctoral-level students in clinical psychology) to identify which of the apps utilized psychological components. App descriptions on the app stores and developer websites were reviewed for any mention of the following: (1) CBT-related terms (e.g., cognitive behavioral therapy, CBT, cognitive restructuring, behavioral activation), (2) ACT-related terms (e.g., acceptance and commitment therapy, ACT, values, accepting thoughts), (3) mindfulness-related terms (e.g., mindfulness, mindfulness-based, present moment, meditation), and (4) general reference to psychology (e.g., psychology, psychological). The two reviewers were instructed to be inclusive at this stage. For example, if an app described a psychological principle but it was not “word-for-word” in our list of possible terms, it was still included (e.g., if an app stated “be in the moment, think about the here and now” it would have been included).

### Psychological Component Checklist

Apps that passed the second stage of screening were downloaded and reviewed by the same two independent reviewers. Apps were reviewed for a minimum of 15 min or until reviewers felt they were able to adequately identify and assess all components (to a maximum of three weeks to unlock any features that became available with use). An 18-item psychological components checklist was developed by the research team using Portelli and Eldred’s checklist^38^ as a starting point and based on the core components typically included in CBT, ACT, and mindfulness interventions for chronic pain and comorbid mental health conditions. Ten of these items were the same or similar to those included in Portelli and Eldred’s checklist^38^ and are commonly included in CBT interventions (e.g., pain diary, sleep hygeine). Six additional items were generated to reflect components that tend to receive greater emphasis in ACT and mindfulness interventions (e.g., values, self-compassion).^48,49^ Given the importance of providing tailored feedback to users^50,51^ and ensuring that apps are considering the culture and diversity of users,^30^ we added two additional items to address the same. A detailed operational description of each item is provided in the Supplementary File 1. The items are listed briefly here: psychoeducation, pain diary, tailored feedback, goal setting, activity pacing, physical activity, sleep hygiene, behavioral activation, cognitive restructuring, coping skills training, relaxation training, mindfulness/present moment awareness, self-compassion, values, acceptance, other ACT principles (e.g., other cognitive defusion skills, self-as-context), social support, and culture/diversity. One point was assigned if at least one function or activity on the app related to a given psychological component, with possible total scores ranging from 0 to 18. Higher scores indicated more psychological components.

### Mobile App Rating Scale

Apps were also evaluated using the MARS. The MARS is a widely used, standardized tool consisting of 23 items that assess apps on five dimensions: engagement, functionality, esthetics, quality of information, and a subjective rating of quality.^45^ Each item is rated on a 5-point scale using specific instructions and examples for what constitutes each score. Higher scores indicate better quality. In line with previous studies,^52,53^ two independent raters (first and third author) used each app for a minimum of 10 min in order to familiarize themselves with the app functionality and user experiences. If any individual item differed by two or more points between reviewers, the reviewers met to discuss differences and come to a consensus. Consistent with previous studies, average ratings were calculated for each of the five dimensions and a final composite score for app quality was calculated by averaging the dimensions of engagement, functionality, esthetics, and quality of information.^34,54,55^

### Statistical Procedure

Our study protocol required two reviewers to make independent judgments regarding whether an app should be included in our analysis, as well as assign scores on a psychological components checklist and the MARS. Because a small number of apps identified in our search had been reviewed in previous publications, reviewers were blinded to these ratings. In the case of disagreements regarding whether an app should be included in our review or how an app should be scored on the psychological components checklist, the two reviewers discussed the disagreement to come to a consensus. On the rare occasion when a consensus could not be reached, the senior author was included in a second round of discussions and consensus was then achieved. Cohen’s kappa was calculated to establish intercoder reliability for the psychological components checklist. Intraclass correlation was calculated to establish intercoder reliability for the MARS. Descriptive statistics were used to present the scores on the psychological components checklist and the MARS.

## Results

### Summary of App Search

**Figure 1.**
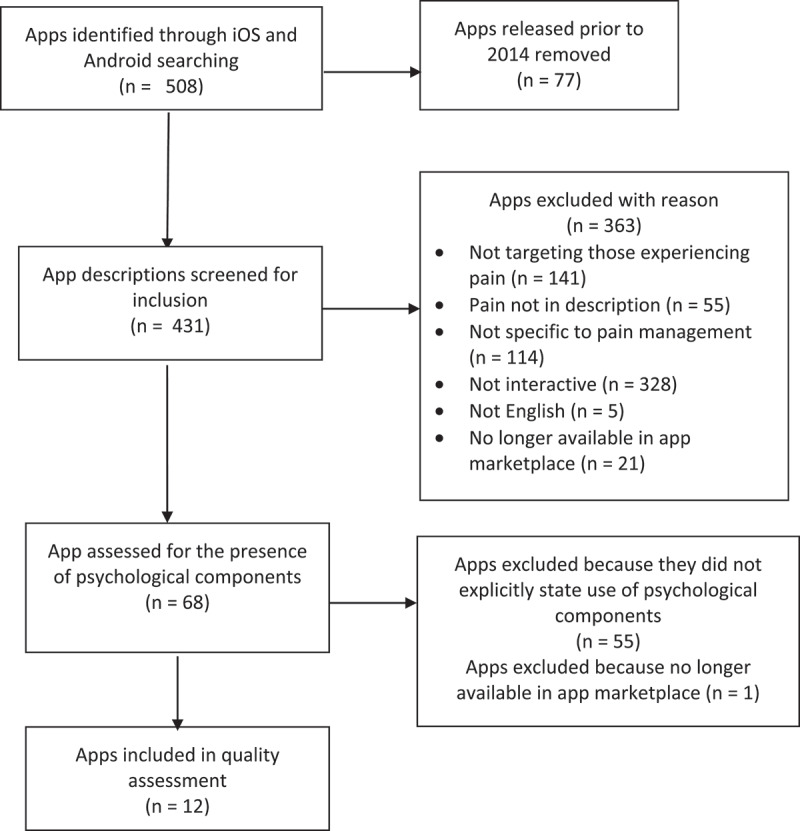
PRISMA (Preferred Reporting Items for Systematic Reviews and Meta-Analyses) flowchart.

A total of 508 apps were identified using our initial search terms (“pain,” “chronic pain,” “pain management”), 68 of which met initial inclusion criteria and went on the the second stage of screening. The specific reasons for apps being excluded are displayed in Figure 1. Thirteen apps had at least one psychological component. One of these apps (Beyond Pain) was removed from the Apple and Google Play stores during article preparation, leaving 12 apps to be included in this review.

### Overview of Pain Apps with Psychological Components

Three of the 12 included apps were available on iPhone only, 1 was available on Android only, and the remaining 8 were available on both platforms. At the time of data collection, all 12 apps were available to download at no cost to the user; however, the majority of these apps (*n* = 8) required the user to “unlock” features via in-app purchases. Key characteristics of the apps are described in Table 1. Nine apps targeted adults, 2 apps targeted adolescents, and 1 app targeted children.

**Table 1.** Description of apps

App (developer)	Target audience	Data privacy and security	Cost	App description	PCC(/18)	MARS (/5)
Curable Pain Relief (Curable Inc.)	Adults	https://www.curablehealth.com/privacy	Full program US$12.81/month	A virtual coach takes users through tailored lessons and exercises, helping them understand the cycle of pain and mind–body interactions	12	4.38
Pathways Pain Relief (Pathways)	Adults	https://www.pathways.health/privacy-policy/	Full program US$69.99/year	A guided pain relief program to provide those experiencing pain with comprehensive pain management skills	12	4.13
Vivify (Vivify AS)	Adults with back and neck pain	https://vivify-app.com/privacy/	US$59/year	A 28-day program consisting of education videos, meditations, exercises, and guided walks	10	4.13
WebMAP Mobile (Seattle Children’s Hospital)	Teens	No information found	Free	A 6-week psychoeducational program teaching teens behavioral and cognitive skills to manage pain and improve their ability to engage in daily activities	9	3.88
Beatpain (Smith & Co Trust)	Adults	https://www.beatpain.app/privacy-policy/<Q32/>	Free	A guided program providing a pain tracker, daily coaching system, and weekly solutions to deal with pain based on the user’s self-identified needs	9	2.25
iBeatPain for Teens (Take The Wind Lda)	Teens	No information found	Free	Provides tips on daily steps that teens can take to get their lives back and return to normal daily functioning	7	2.50
Managing OA Pain (Michael Pincus)	Adults with knee OA	https://appcdn.media/privacy/?app=ManagingOAPain	Free	Offers eight weekly sessions including CBT and self-help techniques for individuals with knee OA. Sessions consist of both video and written instruction	7	2.00
Branch (Ouchie LLC)	Adults	https://branch.health/privacy	Free	Users can track their symptoms, connect with peers, and get advice from medical professionals	6	3.63
Pain Toolkit (Advanced Digital Innovations [UK] Ltd)	Adults	http://pain-sense.co.uk/privacy-policy/	Full program £4.99	A digital version of the Pain Toolkit booklet written by Pete Moore and Dr. Frances Cole that includes 12 self-help tools, a diary, and self-assessment tools	5	2.75
Mayv (Dala Inc.)	Adults	https://app.termly.io/document/privacy-policy/971bc389-09d9-4c09-b556-004db4015144	Full features US$13/month	A suite of pain management tools including an in-app community to allow users to connect and share experiences. A 4-week mind–body program includes deep dives into specific topics (e.g., sleep, nutrition), daily mindfulness practices, and a tool for quick relief	4	3.13
Manage My Pain (ManagingLife Inc.)	Adults	https://managemypainapp.com/privacy-policy	US$4/month for Pro version	Users can record their feeling, medications, and whether they engaged in meaningful activities. Charts of user’s records over time can assist users in recognizing patterns and trends (Pro version provides reports >30 days)	2	3.00
Achy Penguin (For Jack and Jill LLC)	Children	http://forjackandjill.com/privacy.html	Free	This app incorporates techniques such as breathing, relaxation, visualization, and distraction	2	2.38

PCC = Psychological Components Checklist; OA = osteoarthritis.

#### Psychological Components Checklist

The results from the psychological components checklist are presented in Table 1 (scores for each individual item on the checklist are available online in the Supplementary File 2). The average score across all apps was 8.10 out of 18 (SD = 2.77). Both Pathways Pain Relief and Curable Pain Relief received the highest score of 12. Intercoder agreement of psychological components present/absent determined by Cohen’s kappa (κ) was 0.90, indicating excellent agreement between coders. The senior author was involved in resolving 5 of the 12 disagreements, which were easily resolved and predominantly related to an app feature that was difficult to locate.

The number of apps that included each psychological component are listed here from most frequent to least frequent: psychoeducation (*n* = 10), sleep hygiene (*n* = 8), behavioral activation (*n* = 7), coping skills training (*n* = 7), social support (*n* = 7), relaxation training (*n* = 6), mindfulness training (*n* = 5), pain diary (*n* = 5), physical activity (*n* = 5), cognitive restructuring (*n* = 5), acceptance (*n* = 4), activity pacing (*n* = 3), self-compassion (*n* = 3), tailored feedback (*n* = 2), goal setting (*n* = 2), values (*n* = 1), other ACT skills (*n* = 1), and culture/diversity (*n* = 0).

### MARS

On average, apps received a composite quality score of 4.02 out of 5 (SD = 0.32); see Table 1 for individual app scores on the MARS and see Supplementary File 3 for app scores on each item. Manage OA Pain received the lowest composite score of 3.51 and Pathways Pain Relief scored the highest at 4.50. Average subcategory scores across all 12 apps were as follows: functionality (M = 4.18; SD = 0.36), esthetics (M = 4.03; SD = 0.60), quality of information (M = 3.97; SD = 0.33), engagement (M = 3.92; SD = 0.53), and subjective rating of quality (M = 3.18; SD = 0.82). The intraclass correlation for intercoder agreement of MARS scores was 0.91, indicating excellent agreement between coders.

Of note, only 1 of the 12 reviewed apps had undergone a randomized trial (WebMAP).^56^ Two apps (Manage My Pain and Achy Penguin) had been tested or trialed to some extent, and the available evidence suggests that they are feasible and well accepted.^57,58^ An additional two apps (Curable Pain Relief and Pathways Pain Relief) were reported to currently have pilot studies underway. Eight of the 12 apps indicated that a health care professional contributed in some way in their development, such as hosting their own educational modules within the app and/or recommending specific techniques or strategies to be included in the apps.

### Top Rated Apps

Three apps (Curable Pain Relief, Pathways Pain Relief, and Vivify) achieved the highest scores on the MARS while also including the highest number of psychological components.

Curable Pain Relief targets adults experiencing chronic pain. The app features a virtual coach who presents information and techniques in brief segments (ranging from 5 to 20 min). The virtual coach communicates with the user in a text-based, interactive format. Users are asked to select from one of four different categories (education, meditation, writing, or brain training) and are then offered an exercise within that domain (e.g., psychoeducation about pain catastrophizing, reframing negative thoughts). Future content is individually tailored based on the user’s feedback. Guided breathing exercises can be selected as needed. A range of workshops, recovery stories, expert interviews, and options to join a support community are also offered.

According to the app website, development was overseen by a scientific advisory team and individuals with lived experience of chronic pain. An ongoing study is being conducted by Curable to determine app efficacy, and their website provides non-peer-reviewed preliminary evidence demonstrating improved quality of life, reduced anxiety, and physical pain relief among users (https://www.curablehealth.com/science). It is free to download, but an annual subscription is required to access all components.

Pathways Pain Relief offers a guided, four-step program for adults experiencing chronic pain. Step 1 provides “essential” pain relief techniques (e.g., pain meditation, visualization). Step 2 introduces pain management strategies (e.g., graded exposure, pacing). Step 3 focuses on improving self-care, acceptance, and gratitude. Step 4 focuses on a range of mindfulness and meditation practices, including compassion-based approaches. Activities are unlocked as users move through the program, but the app can also be utilized in a self-directed manner. The app description states it was created by scientists and patients with pain. To date, there is no research to support the efficacy of this app. Pathways is free to download, though in order to access all included programs, a monthly, yearly, or lifetime subscription is required.

Vivify provides users with chronic back or neck pain with a 28-day video-based program consisting of pain education, meditations, exercises, and guided walks. The program is presented by two people with lived experience of chronic pain. Each day, an education session (e.g., biopsychosocial model), a meditation, an exercise (e.g., squats), and an audio-guided walk with a topic (e.g., challenging unskillful thought patterns) are offered. Following the 28-day program, users continue to have access to all activities. The app description states that Vivify was developed by health care professionals who work with individuals with chronic lower back and neck pain. To date, there is no research to support the efficacy of this app, and a monthly or yearly fee is required.

## Discussion

With one in four Canadians experiencing chronic pain^1^ and over 85% of Canadians having a mobile phone subscription,^59^ apps can be a convenient and accessible tool to support patients in managing their pain in daily life. The present review offers valuable insights into the extent to which core aspects of empirically supported psychological treatments are being integrated into pain management apps available in Canada, as well as a validated assessment of app quality. Our search identified 508 pain management apps that have been released since 2014, but only 12 included one or more component of cognitive behavioral, mindfulness, or acceptance-based therapeutic approaches. Of these 12 apps, the majority targeted adults and utilized CBT techniques. Only 5 offered mindfulness-based content, and 2 offered some ACT techniques. We found evidence for growing efforts to include patients and health care providers in the design of pain management apps and to build an evidence base for app efficacy. However, there remains limited to no empirical evidence to support the efficacy of even the highest rated apps in this review.

### Psychological Components within Pain Management Apps

Though previous reviews have focused on pain self-mangement strategies in general, or CBT in particular, there has been little attention given to acceptance- and mindfulness-based approaches in pain management apps. Despite specifically targetting these latter approaches in our search and evaluation criteria, CBT remains the most commonly included psychological approach within pain management apps. Yet even CBT programming was highly variable across apps and tended to focus on specific CBT techniques rather than offering a comprehensive CBT intervention that is more typical of in-person and web-based formats. Similar to Devan and colleagues’^30^ findings, apps that provided assistance with goal setting were surprisingly few and far between, and no app addressed issues related to culture or diversity.

Despite a large body of literature supporting the efficacy of ACT and mindfulness approaches for the management of chronic pain,^7–11^ no app included in the current review provided a comprehensive ACT or mindfulness-based approach. Only one app offered acceptance strategies (Curable), one app provided some values work (Vivify), and one app (Curable) offered a cognitive defusion exercise, which are typically considered core components of ACT for chronic pain. Similarly, mindfulness approaches received only modest attention in currently available pain management apps, with five apps including mindfulness practices (Curable, Pathways, Vivify, Branch, Mayv) and three apps offering self-compassion practices (Curable, Pathways, Vivify).

### Quality of Pain Management Apps

In addition to a detailed review of psychological components, the current study employed the standardized MARS tool to assess app quality. Similar to past reviews of pain management apps,^30,31,34^ apps performed best on the functionality and esthetics subcategories of the MARS. However, the average app quality (M = 4.02 out of 5) was notably higher than reported in previous reviews, which have published scores ranging from 3.13 to 3.76.^30,31,34,37^ The higher overall score for app quality in our review was likely driven by higher scores on engagement (M = 3.92 out of 5) and information (M = 3.97 out of 5) compared to past reviews (which ranged from 2.81 to 3.21 for engagement and 2.48 to 3.64 for information).^30,31,34,37^ Higher engagement scores may have been due to our exclusion of apps that did not have interactive components. Higher information scores may have been related to the exclusion of apps that did not contain any psychological components. It is unlikely that our MARS ratings were systematically inflated because our ratings for specific apps were similar to those published in past reviews (and our raters were blinded to these reviews). For example, Devan et al.^30^ gave Curable Pain Relief an overall score of 4.54 and information score of 4.5 (our scores were 4.49 and 4.3, respectively).

This review also gleaned important information regarding app development and available research base to support app efficacy. Only one app (WebMAP) has been tested within a randomized trial.^56^ To the best of our knowledge, no randomized trial designs have been used to test the efficacy of the remaining 11 apps in this review. However, our findings suggest that the field is moving in the right direction. Within Devan and colleagues’^30^ review, only 52% of included apps had involved health care practitioners, and only 21% had been trialed to some extent. In the current review, 8 apps (66%) included health care practitioners in the development of the app content, and 5 apps (42%) had been tested or trialed to some extent (WebMAP, and Achy Penguin, and Manage My Pain)^56–58^ or reportedy have pilot studies underway (Curable and Pathways).

### Recommendations for Clinicians

Three apps (Curable Pain Relief, Pathways Pain Relief, or Vivify) emerged as having the highest scores on both the MARS and the psychological checklist, and all were designed for adults with chronic pain. Consistent with the other apps in this review, all three were available in both the U.S. and Canadian app stores. It is important to note that despite receiving high scores on quality and ease of use on the MARS, none of these three apps had undergone independent scientific review at the time of data collection. Nonetheless, they included at least ten different psychological components derived from empirically supported treatments for chronic pain, depression, and anxiety. These were largely grounded in CBT, with some additional mindfulness- and acceptance-based techniques. Each app had its own unique strengths, which may hold appeal for different users. For example, an interesting approach in Vivify was the use of “guided walks,” where participants learn about a theme during the walk (e.g., self-efficacy), which is a unique strategy to simultaneously foster behavioral activation, physical activity, and psychoeducation. Curable scored highest on engagement and esthetics within the MARS. Pathways had a notably wide range of video-based guided meditations and relaxation training, physiotherapy exerices, yoga, and foam rolling sessions.

Options are currently limited for children and adolescents. Achy Penguin appears to be the only app available in Canada that is suitable for young children (specifically those with acute pain), and it has undergone rigorous usability testing through a formal partnership between parents and pain scientists.^58^ WebMAP stood out as being particularly promising for adolescents based on its high MARS score in the current study, recent encouraging results for patient global impression of change,^56^ and its provision of a structured, comprehensive, 6-week CBT program. Indeed, it was one of few apps in our review that followed a more typical in-person or Internet-based delivery format in which users are systematically guided through pain management education and skills in a step-wise, interactive manner.

### Study Limitations

A challenge in conducting any standardized review of smartphone apps for chronic health conditions is that the Android and iOS app marketplaces are highly dynamic, constantly evolving entities. For example, during our article preparation, one app changed its name (Branch, previously known as Ouchie) and another was removed from app stores (Beyond Pain). Although we ensured that psychological checklist and MARS scores were accurate at time of article submission, the accuracy may shift over time, in unpredictable ways. It is also possible that, despite using a very hands-on review of each app, we may have missed certain app content or functions. In the absence of any standardized tools to assess psychological content, we used a checklist designed for the purposes of our study. This checklist used a dichotomous rating system to determine whether a psychological component was present; however, this did not account for the breadth and depth in which a topic was covered. Thus, our assessment strategy may have been somewhat biased against apps that did a high-quality job of delivering only a small number of pain management strategies. For example, Manage My Pain was designed to be a pain tracking tool to help patients identify factors that worsen or improve pain (i.e., not a comprehensive pain management tool). Thus, the low score on our psychological components checklist should not be interpreted as evidence against its potential value for its intended purpose. Additionally, our search did not capture apps released prior to 2014 (but have since been updated), apps restricted to specific clinics, apps not available in Canada, or apps in languages other than English. Finally, by limiting the search to apps targeting pain more generally (i.e., excluding pain apps related to a specific condition), this review has likely omitted many condition-specific apps and other mHealth solutions (e.g., online pain management programs) that may be useful for clinicians and patients alike.

### Future Directions

Broadly, the current review highlights the pressing need for rigorous, independent scientific development and review of pain management apps that deliver psychological strategies. In particular, no apps provided a comprehensive ACT or mindfulness approach that targets people with chronic pain, and only three pain apps target children and adolescents. Given the high adoption of Internet-enabled devices among youth,^60^ app-based interventions are a promising, yet underexplored, approach to pain treatment in younger age groups. Attention also needs to be given to how apps might be adapted for use among older adults, who experience the highest rates of chronic pain.^61^ Finally, research has shown that low-income, minority, and stigmatized groups lack equal access to pain services, and this problem extends to the domain of pain management apps.^62,63^ Future app development and related research should determine what adaptations and tailoring can optimally influence access and engagement with mHealth across diverse populations.^64^

Many pain management apps (including the top three identified in the current study) charge users a monthly or annual fee to access the full suite of in-app features. Favorable cost-effectiveness studies are needed to help justify a shift in the current payment structure. Ideally, the cost for empirically supported mHealth apps could be diverted away from patients and onto private and public health care plans. Research aimed at enhancing patient engagement with mHealth apps is also of the utmost importance moving forward.^57,65^ Even if an app’s efficacy has been shown within an RCT, it will not be effective unless patients continue to engage with applications and adhere to strategies to manage their pain over time. Despite the fact that the current review saw higher scores on the MARS subscale for engagement compared to previous reviews, this was still among the lowest scored subscales, highlighting it as an area for improvement. Researchers should identify ways to optimize patient engagement. For example, researchers may examine what additional interactive components bolster patients’ feelings of accountability and social support. Integration of activity monitors and sensors, as well as two-way messaging, may help enhance patient engagement while also improving clinicians’ abilities to remotely monitor patient behavior and provide real-time feedback on their progress.

#### Integration into Clinical Practice

If developed to meet both clinician and patient needs, apps can be used as a supplementary tool in pain management to engage patients, enhance care, and potentially reduce health care costs. This successful integration into clinical practice can be achieved by developing apps with clinical implementation in mind. A major challenge in integrating mHealth in chronic pain treatment is inconsistent use of the data generated from the mHealth technology by the care team.^15^ In fact, patient data derived from mobile devices are rarely incorporated within electronic medical records.^15,66^ To ensure that mHealth interventions for chronic pain meet end-user needs and are effectively translated into clinical practice, it will be critical to continue to include end-users (e.g., physicians, nurses, patients, family members) throughout the phases of development and evaluation.^58^ By engaging pain psychology experts, apps will be more likely to align with current best practice guidelines and evidence-based psychological approaches for treating chronic pain.

#### App Prescription

From both patient and provider perspectives, navigating the world of smartphone apps for managing chronic pain remains a challenge, particularly when seeking apps that include evidence-based psychological components. The app descriptions provided in Table 1 and the supplemental materials (which list the specific psychological components in each app, such as sleep hygiene and activity pacing) can help clinicians make rapid, informed, and tailored app recommendations to their patients. However, as mHealth continues to advance and become increasingly woven into pain management, automated decision aids for app prescription are needed. To be most effective, these decision aids will need to take into account the full picture of a patient’s physical, psychological, and social functioning. This type of tool could be integrated into current stepped care approaches for chronic pain.^67^ For some patients, an app could be a convenient, cost-saving waitlist intervention or even alternative to intensive group or individual therapy, and for others it might be more appropriate as a companion or adjunct to a comprehensive pain treatment plan.

Given the evolving nature of mHealth apps, even those tested in a randomized controlled trial may no longer be evidence-based in the years following evaluation (e.g., because of new updates that remove or alter features that were driving the efficacy). To improve the validity and utility of mHealth evaluations, researchers should identify how specific CBT, mindfulness, and ACT app components can optimally influence pain management (i.e., what components are best suited for an app-based platform and how can they be best presented to facilitate user engagement).

### Conclusions

The demand for psychosocial support in managing chronic pain and associated mental health concerns has long outweighed the support that is available to patients.^64^ mHealth technolologies, including smartphone applications for pain self-management, have been identified as holding great promise as scalable interventions that could be integrated, or even help shape, service delivery models.^64^ The current review identified 12 apps available to residents of Canada that have potential to improve patient health and well-being via empirically supported CBT, ACT, and mindfulness-based techniques. However, before any such app achieves widespread adoption, researchers and developers must address the growing calls for better quality control and formal scientific evaluation of available apps, equitable access, and, more broadly, how these newer technologies can contribute to the evolution of best practices in pain care.^68^

## Supplementary Material

Supplemental Material
