# Supplementary material for: Do pain management apps use evidence-based psychological components? A systematic review of app content and quality
Source: Can J Pain. 2022 Jun 3;6(1):33–44. doi: 10.1080/24740527.2022.2030212 (PMC9176230; doi:10.1080/24740527.2022.2030212)
Supplement: Supplemental Material [file UCJP_A_2030212_SM1408.docx]

Supplementary File 1. Psychological Components Checklist

**Instructions: Each app should be downloaded and used for at least 10 minutes. All functions of the app should be explored before scoring. Apps should be given a score of 1 point for each item featured within the pain, for possible score of 0 – 19.**

**NAME OF APP:**

| **Psychological Component** | **Description of Psychological Component** | **Score**  **(1 vs. 0)** |
| --- | --- | --- |
| **(1) Psychoeducation** | Presents information regarding one or more of the following: biopsychosocial model of pain; gate control theory of pain; CBT model; ACT model (e.g., hexaflex, triflex) |  |
| **(2) Pain Diary** | Allows patient to track pain and psychosocial factors (e.g., mood, ways of coping, situations) that may relate to pain |  |
| (3) Tailored Feedback | Provides patients with tailored feedback on factors that may be impacting their pain (e.g., based on pain diary and/or other questionnaires) |  |
| (4) Goal Setting | Helps patients create “SMART” goals (with or without focus on values); Problem solving |  |
| **(5) Activity Pacing** | Helps patient plan daily activities/physical activity so as not to ‘overdo it’ |  |
| **(6) Physical Activity** | Guided instructions and/or videos for aerobic (e.g., walking, swimming) or anaerobic (strengthening, stretching, yoga, tai chi, pilates) exercises |  |
| **(7) Sleep hygiene** | Provides education and/or strategies to improve sleep |  |
| **(8) Behavioural Activation** | Encourages patients to engage in activities that will be reinforcing and/or enjoyable (e.g., pleasant activity scheduling) |  |
| **(9) Cognitive Restructuring** | Monitoring, labelling, and challenging negative thoughts (e.g., catastrophizing); thought records; education about cognitive distortions; underlying attitudes/beliefs about pain |  |
| **(10) Coping Skills Training** | Identifying and reducing maladaptive coping (e.g., avoidance, denial, venting) and enhancing adaptive coping (e.g., positive self-statements, positive reappraisal, finding meaning, distraction, virtual reality) |  |
| **(11) Relaxation Training** | Education, demonstrations, activities to support diaphragmatic breathing, guided imagery, passive or progressive muscle relaxation, hypnotherapy) |  |
| **(12) Values** | Education and activities to help identify personal values (e.g., Bull’s eye, Values cards) |  |
| **(13) Acceptance** | Education and activities to encourage acceptance (e.g., pushing away paper, passengers on a bus, struggle switch) |  |
| **(14) Mindfulness/Present Moment** | Education, demonstrations, activities to support mindfulness (e.g., body scan, sitting meditation, walking meditation, leaves on a stream, dropping the anchor) |  |
| **(15) Other ACT skills (i.e. cognitive defusion above and beyond mindfulness, self as context)** | Education and activities to support cognitive defusion (e.g., noticing, naming the story, neutralizing) that are above and beyond mindfulness/meditation practices. Notice you are noticing. |  |
| **(16) Self-compassion** | Education and exercises to support self-compassion (e.g., kind hand) |  |
| **(17) Social Support** | Education and activities to help patients build social support (online or offline); enhance adaptive support seeking and assertive communication, reduce solicitous behaviour; builds peer support (e.g., offers platform to connect with similar others) |  |
| **(18) Culture/Diversity** | Addresses role of culture, ethnicity, gender, SES, sexual orientation that impacts coping with chronic pain |  |

Supplementary File 2. Quality Checklist

All apps were rated as either having the specified psychological components present (1) or absent (0)

|  | **Curable Pain Relief** | **Pathways Pain Relief** | **Vivify** | **Beatpain** | **WebMAP Mobile** | **iBeat**  **Pain for Teens** | **Managing OA Pain** | **Branch** | **Pain Toolkit** | **Mayv** | **Achy Penguin** | **Manage My Pain** | **Total # using this item (/12)** |
| --- | --- | --- | --- | --- | --- | --- | --- | --- | --- | --- | --- | --- | --- |
| **Psychoeducation** | 1 | 1 | 1 | 1 | 1 | 1 | 1 | 1 | 1 | 1 | 0 | 0 | 10 |
| **Pain Diary** | 0 | 1 | 0 | 1 | 1 | 1 | 0 | 1 | 0 | 0 | 0 | 1 | 6 |
| **Tailored Feedback** | 1 | 0 | 0 | 1 | 0 | 0 | 0 | 0 | 0 | 0 | 0 | 0 | 2 |
| **Goal Setting** | 0 | 0 | 0 | 0 | 1 | 1 | 0 | 0 | 0 | 0 | 0 | 0 | 2 |
| **Activity Pacing** | 0 | 0 | 0 | 0 | 1 | 0 | 1 | 0 | 1 | 0 | 0 | 0 | 3 |
| **Physical Activity** | 0 | 1 | 1 | 1 | 0 | 0 | 0 | 1 | 0 | 1 | 0 | 0 | 5 |
| **Sleep Hygiene** | 1 | 1 | 1 | 1 | 1 | 1 | 1 | 0 | 1 | 0 | 0 | 0 | 8 |
| **Behavioural Activation** | 1 | 1 | 0 | 1 | 1 | 1 | 1 | 0 | 1 | 0 | 0 | 1 | 8 |
| **Cognitive Restructuring** | 1 | 1 | 1 | 0 | 1 | 0 | 1 | 0 | 0 | 0 | 0 | 0 | 5 |
| **Coping Skills Training** | 1 | 1 | 1 | 1 | 0 | 1 | 1 | 1 | 0 | 0 | 1 | 0 | 8 |
| **Relaxation Training** | 1 | 1 | 0 | 1 | 1 | 0 | 1 | 0 | 0 | 1 | 1 | 0 | 7 |
| **Values** | 0 | 0 | 1 | 0 | 0 | 0 | 0 | 0 | 0 | 0 | 0 | 0 | 1 |
| **Acceptance** | 1 | 1 | 1 | 0 | 0 | 0 | 0 | 0 | 1 | 0 | 0 | 0 | 4 |
| **Mindfulness/ Present Moment** | 1 | 1 | 1 | 0 | 0 | 0 | 0 | 1 | 0 | 1 | 0 | 0 | 5 |
| **Other ACT skills** | 1 | 0 | 0 | 0 | 0 | 0 | 0 | 0 | 0 | 0 | 0 | 0 | 1 |
| **Self-Compassion** | 1 | 1 | 1 | 0 | 0 | 0 | 0 | 0 | 0 | 0 | 0 | 0 | 3 |
| **Social Support** | 1 | 1 | 1 | 1 | 1 | 1 | 0 | 1 | 0 | 0 | 0 | 0 | 7 |
| **Culture/ Diversity** | 0 | 0 | 0 | 0 | 0 | 0 | 0 | 0 | 0 | 0 | 0 | 0 | 0 |
| **Total Score (/18)** | **12** | **12** | **10** | **9** | **9** | **7** | **7** | **6** | **5** | **4** | **2** | **2** |  |

Supplementary File 3. Mobile Application Rating Scale (MARS)

All apps were rating on the following items on a scale of 1 – 5 (with lower scores indicating poorer quality)

|  | **Pathways** | **Curable** | **Vivify** | **Mayv** | **Branch** | **WebMAP Mobile** | **iBeat Pain for Teens** | **Achy Penguin** | **Beat pain** | **Pain Toolkit** | **Manage My Pain** | **Managing OA Pain** |
| --- | --- | --- | --- | --- | --- | --- | --- | --- | --- | --- | --- | --- |
| **A. Engagement** |  |  |  |  |  |  |  |  |  |  |  |  |
| Entertainment | 4.00 | 4.50 | 4.50 | 4.50 | 5.00 | 4.00 | 4.00 | 4.50 | 4.00 | 2.50 | 2.00 | 3.50 |
| Interest | 5.00 | 5.00 | 4.50 | 4.00 | 4.50 | 4.50 | 4.00 | 4.50 | 4.00 | 3.00 | 3.00 | 3.50 |
| Customization | 4.00 | 4.00 | 3.50 | 3.00 | 4.00 | 2.50 | 4.50 | 1.50 | 3.00 | 1.50 | 3.50 | 1.50 |
| Interactivity | 4.00 | 4.50 | 3.50 | 4.50 | 4.00 | 4.00 | 4.00 | 4.00 | 3.50 | 3.50 | 3.50 | 2.50 |
| Targeted | 5.00 | 5.00 | 4.50 | 5.00 | 4.50 | 4.50 | 5.00 | 5.00 | 4.50 | 4.50 | 4.50 | 4.50 |
| Engagement mean score | **4.40** | **4.60** | **4.10** | **4.20** | **4.40** | **3.90** | **4.30** | **3.90** | **3.80** | **3.00** | **3.30** | **3.10** |
| **B. Functionality** |  |  |  |  |  |  |  |  |  |  |  |  |
| Performance | 5.00 | 4.50 | 5.00 | 5.00 | 4.00 | 4.00 | 4.00 | 3.50 | 4.00 | 4.00 | 5.00 | 3.50 |
| Ease of use | 3.00 | 4.00 | 4.50 | 4.00 | 3.00 | 3.50 | 4.00 | 4.50 | 4.50 | 4.00 | 3.50 | 4.50 |
| Navigation | 5.00 | 4.00 | 5.00 | 4.00 | 3.50 | 4.50 | 4.00 | 4.00 | 4.50 | 4.00 | 3.50 | 4.50 |
| Gestural design | 5.00 | 5.00 | 4.50 | 4.50 | 3.00 | 4.00 | 3.50 | 4.00 | 4.50 | 4.50 | 4.00 | 5.00 |
| Functionality mean score | **4.50** | **4.38** | **4.75** | **4.38** | **3.38** | **4.00** | **3.88** | **4.00** | **4.38** | **4.13** | **4.00** | **4.38** |
| **C. Aesthetics** |  |  |  |  |  |  |  |  |  |  |  |  |
| Layout | 4.50 | 4.50 | 4.50 | 4.00 | 4.00 | 4.00 | 3.50 | 4.00 | 4.00 | 4.50 | 4.00 | 3.50 |
| Graphics | 5.00 | 5.00 | 5.00 | 4.50 | 4.50 | 4.00 | 3.50 | 3.50 | 4.00 | 4.00 | 3.50 | 2.00 |
| Visual appeal | 4.50 | 4.50 | 5.00 | 5.00 | 4.50 | 4.50 | 3.50 | 4.00 | 3.00 | 3.00 | 3.00 | 3.00 |
| Aesthetics mean score | **4.67** | **4.67** | **4.83** | **4.50** | **4.33** | **4.17** | **3.50** | **3.83** | **3.67** | **3.83** | **3.50** | **2.83** |
| **D. Information** |  |  |  |  |  |  |  |  |  |  |  |  |
| Accuracy of app description | 5.00 | 5.00 | 4.50 | 4.00 | 5.00 | 5.00 | 4.50 | 4.50 | 4.50 | 4.50 | 4.50 | 4.50 |
| Goals | 5.00 | 5.00 | 4.50 | 4.50 | 5.00 | 5.00 | 4.50 | 4.50 | 3.50 | 4.00 | 4.50 | 3.50 |
| Quality of information | 5.00 | 5.00 | 4.50 | 4.00 | 4.00 | 5.00 | 3.50 | 3.00 | 4.50 | 4.50 | 5.00 | 4.50 |
| Quantity of information | 4.50 | 4.00 | 3.50 | 3.50 | 4.00 | 4.00 | 3.50 | 3.00 | 4.00 | 4.00 | 4.50 | 4.00 |
| Visual information | 4.00 | 4.00 | 4.00 | 3.50 | 4.50 | 4.00 | 4.00 | 4.00 | 4.00 | 4.00 | 4.00 | 4.00 |
| Credibility | 3.00 | 3.00 | 3.00 | 1.00 | 3.00 | 3.50 | 3.00 | 4.00 | 1.00 | 3.00 | 3.00 | 2.00 |
| Evidence base | N/A | N/A | N/A | N/A | N/A | 3.00 | N/A | 3.00 | N/A | N/A | 3.00 | N/A |
| Information mean score | **4.42** | **4.33** | **4.00** | **3.42** | **4.25** | **4.21** | **3.83** | **3.71** | **3.58** | **4.00** | **4.07** | **3.75** |
| **E. App subjective quality** |  |  |  |  |  |  |  |  |  |  |  |  |
| Would you recommend this app | 5.00 | 5.00 | 4.50 | 3.50 | 3.50 | 4.50 | 2.50 | 3.00 | 2.50 | 3.00 | 3.00 | 1.50 |
| How many times would you use this app in the next 12 months | 4.50 | 4.50 | 4.00 | 3.50 | 4.50 | 4.00 | 2.50 | 2.50 | 3.00 | 3.00 | 3.50 | 2.50 |
| Would you pay for this app? | 3.00 | 4.00 | 4.00 | 2.00 | 3.00 | 3.00 | 2.00 | 1.00 | 1.00 | 2.00 | 2.00 | 1.00 |
| Your overall star rating | 4.00 | 4.00 | 4.00 | 3.50 | 3.50 | 4.00 | 3.00 | 3.00 | 2.50 | 3.00 | 3.50 | 3.00 |
| App subjective quality score | **4.13** | **4.38** | **4.13** | **3.13** | **3.63** | **3.88** | **2.50** | **2.38** | **2.25** | **2.75** | **3.00** | **2.00** |
| **App quality mean score (average of subsection A – D)** | **4.50** | **4.49** | **4.42** | **4.12** | **4.09** | **4.07** | **3.88** | **3.86** | **3.86** | **3.74** | **3.72** | **3.51** |
